# Preconception Maternal Iodine Status Is Positively Associated with IQ but Not with Measures of Executive Function in Childhood

**DOI:** 10.1093/jn/nxy054

**Published:** 2018-05-15

**Authors:** Sian M Robinson, Sarah R Crozier, Elizabeth A Miles, Catharine R Gale, Philip C Calder, Cyrus Cooper, Hazel M Inskip, Keith M Godfrey

**Affiliations:** 1 Medical Research Council (MRC) Lifecourse Epidemiology Unit; 2 Human Development and Health Academic Unit, Faculty of Medicine, University of Southampton, Southampton, United Kingdom; 3 National Institute for Health Research (NIHR) Southampton Biomedical Research Centre, University of Southampton and University Hospital Southampton National Health Service (NHS) Foundation Trust, Southampton, United Kingdom; 4 Centre for Cognitive Ageing and Cognitive Epidemiology, Department of Psychology, University of Edinburgh, Edinburgh, United Kingdom; 5 National Institute for Health Research (NIHR) Musculoskeletal Biomedical Research Unit, University of Oxford, Nuffield Orthopaedic Centre, Oxford, United Kingdom

**Keywords:** iodine, development, cognition

## Abstract

**Background:**

Adverse effects of severe maternal iodine deficiency in pregnancy on fetal brain development are well-established, but the effects of milder deficiency are uncertain. Most studies examine iodine status in pregnancy; less is known about iodine nutrition before conception.

**Objective:**

We examined relations between maternal preconception iodine status and offspring cognitive function, within a prospective mother-offspring cohort.

**Methods:**

Maternal iodine status was assessed through the use of the ratio of iodine:creatinine concentrations (I/Cr) in spot urine samples [median (IQR) period before conception 3.3 y (2.2–4.7 y)]. Childhood cognitive function was assessed at age 6–7 y. Full-scale IQ was assessed via the Wechsler Abbreviated Scale of Intelligence, and executive function through the use of tests from the Cambridge Neuropsychological Test Automated Battery (CANTAB). Analyses (*n* = 654 mother-child dyads) were adjusted for potential confounders including maternal intelligence, education, and breastfeeding duration.

**Results:**

The median (IQR) urinary iodine concentration was 108.4 µg/L (62.2–167.8 µg/L) and the I/Cr ratio 114 µg/g (76–164 µg/g). The preconception I/Cr ratio was positively associated with child IQ, before and after adjustment for potential confounding influences [β = 0.13 (95% CI: 0.04, 0.21)/SD, *P *= 0.003]. 8.9% of women had a preconception urinary I/Cr ratio <50 µg/g; compared with those with an I/Cr ratio ≥150 µg/g, the IQ of their offspring was 0.49 (95% CI: 0.79, 0.18) SD lower. There were no associations with the executive function outcomes assessed via CANTAB, before or after adjustment for confounders.

**Conclusion:**

The positive association between iodine status before conception and child IQ provides some support for demonstrated links between low maternal iodine status in pregnancy and poorer cognitive function reported in other studies. However, given the negative effects on school performance previously observed in children born to iodine-deficient mothers, the lack of associations with measures of executive function in the present study was unexpected. Further data are needed to establish the public health importance of low preconception iodine status.

## Introduction

Iodine is an essential nutrient, required for the synthesis of thyroid hormones, which are known to be critical for cellular metabolism, growth, psychomotor and physical development, and function at all stages of life (1). In pregnancy, the increased synthesis of thyroid hormones, essential for optimal fetal neurodevelopment, increases iodine requirements (2). The fetus is known to be very vulnerable to the effects of iodine deficiency; the effects of severe deficiency on fetal brain development and the risk of cretinism in childhood are well-established (2, 3). However, there is a growing body of evidence that links milder forms of thyroid dysfunction to adverse pregnancy outcomes (4), suggesting that there may be important effects of mild or moderate maternal iodine deficiency on fetal development. At present, there is a lack of good evidence, and any effects on the cognitive function of children born to mildly deficient women are not well understood (2).

In recent years, the United Kingdom has been categorized as mildly iodine-deficient (5), although, based on new data from the UK National Diet and Nutrition Survey (NDNS), its status is now considered to be adequate as all groups studied in the NDNS met the WHO criteria for adequate iodine intake [median urinary iodine concentration (UIC) within the range 100–299μg/L and <20% of samples <50μg/L (6)], including women of childbearing age (7). But it is a concern that high rates of mild to moderate iodine deficiency have been reported in other contemporary UK studies [ranging between 51% in young girls (8), and 40% (9) to 53% (10) among pregnant women], suggesting that deficiency may still be a problem in some subgroups (5). There is no UK recommendation to increase iodine intake in pregnancy, set on the premise that most women have an adequate status that will enable them to meet the additional iodine demands of pregnancy without supplementation (11). However, the marked increase in maternal production of thyroxine in early gestation, at a time when the fetus is wholly dependent on the maternal supply to support normal brain development (12, 13), means that maternal iodine status needs to be sufficient before conception to support this increase. And it is a therefore a concern that low iodine status may be common in women of childbearing age.

These concerns have been heightened by findings of recent studies from developed populations, including the United Kingdom; low maternal iodine status in pregnancy has been linked to poorer cognitive function (14) and poorer school performance in children (15), and associations have been shown between low maternal iodine intakes in pregnancy and language delay and behavior problems in offspring (16). However, to our knowledge, the role of preconception iodine status as an influence on fetal brain development has not been evaluated. Analyzing preconception data from a general population sample of women, we describe maternal preconception iodine status, and examine how it relates to the measured cognitive function of their children when aged 6–7 y.

## Methods

### 

#### Southampton Women's Survey

The Southampton Women's Survey (SWS) is a prospective study of mothers and children (17). For the study, 12,583 nonpregnant women, aged 20–34 y, were interviewed at home; diet and lifestyle were assessed. Of the women interviewed, 3158 became pregnant within the period of the study and had a live singleton infant. These women were followed up in pregnancy; their children have been assessed in ongoing follow-up studies (17). The SWS was approved by the Southampton and South West Hampshire Local Research Ethics Committee (307/97, 153/99w, 005/03/t, 06/Q1702/104, and 10/H0504/30); written informed consent was obtained from all participants. Details of maternal educational attainment (defined in 6 groups according to highest academic qualification) were obtained at the preconception interview. Height and weight were measured, and used to calculate BMI (kg/m^2^). Maternal smoking status was recorded before conception and in pregnancy. Maternal diet was assessed by an administered FFQ, before conception and in early and late pregnancy (18, 19), to record the average consumption of 100 foods over the preceding 3 mo. Food iodine intake was estimated via national food composition data (18, 20). Detailed information on supplement use was collected; supplementary iodine intakes were calculated with the use of manufacturers’ composition data, together with participants’ reported supplement frequency, dose, and duration of use. At birth, the infant was weighed; gestational age was determined via a computerized algorithm based on menstrual data or by ultrasound assessment of fetal anthropometry in early pregnancy. The infants were visited at the ages of 6, 12, and 24 mo; duration of breastfeeding was defined according to the date of the last feed reported at these visits (21).

#### Assessment of iodine status

A single spot urine sample was provided by each SWS woman at clinic visits, after their initial interviews, in the period before conception. The samples were collected at a median of 3.3 y (IQR: 2.2–4.7 y) before conception. The time of day was recorded. Urine samples were frozen and stored at –80°C from the date of collection until being thawed and assayed for iodine and creatinine contents in 2016 by the Trace Element Unit, Southampton General Hospital, Southampton, United Kingdom. Urinary iodine and creatinine concentrations are stable during prolonged frozen storage (14). UIC was assessed with the use of inductively coupled plasma-MS (NexION 300D, PerkinElmer); creatinine concentrations were determined by a Beckman Coulter AU5800 with the use of the Jaffe reaction. UIC was measured in duplicate using rhodium as an internal standard (VWR International). Samples were analyzed against a urine calibration curve with the addition of 0, 1, 2, 5, and 10 µmol I/L (potassium iodide, Fisher Chemicals). Samples for calibration, test, and quality control were diluted 1:15 with diluent containing 0.3% ammonia, 0.4 g ethylenediaminetetraacetic acid disodium salt dehydrate, and 1.2 g ammonium dihydrogen orthophosphate/L (Fisher Chemicals). Results were verified by measurement of certified reference material, Seronorm Trace Elements Urine Levels 1 and 2 (Sero, Norway). Within-run precision was 2.17% (CV) at 0.66 µmol/L for Seronorm Urine Level 1 and 1.21% at 2.30 µmol/L for Seronorm Urine Level 2. Between-run precision was 7.05% (CV) at 0.66 µmol/L for Seronorm Urine Level 1 and 2.20% at 2.30 µmol/L for Seronorm Urine Level 2. To correct measures of iodine concentration for differences in urine volume, an iodine:creatinine ratio (I/Cr) was calculated for each participant; this measure of iodine status was used in all statistical analyses. However, in order to compare our study with new iodine status data available for a national sample of women of childbearing age (7), we also present descriptive data on UIC.

#### Assessment of cognitive function at 6–7 y of age

**FIGURE 1:**
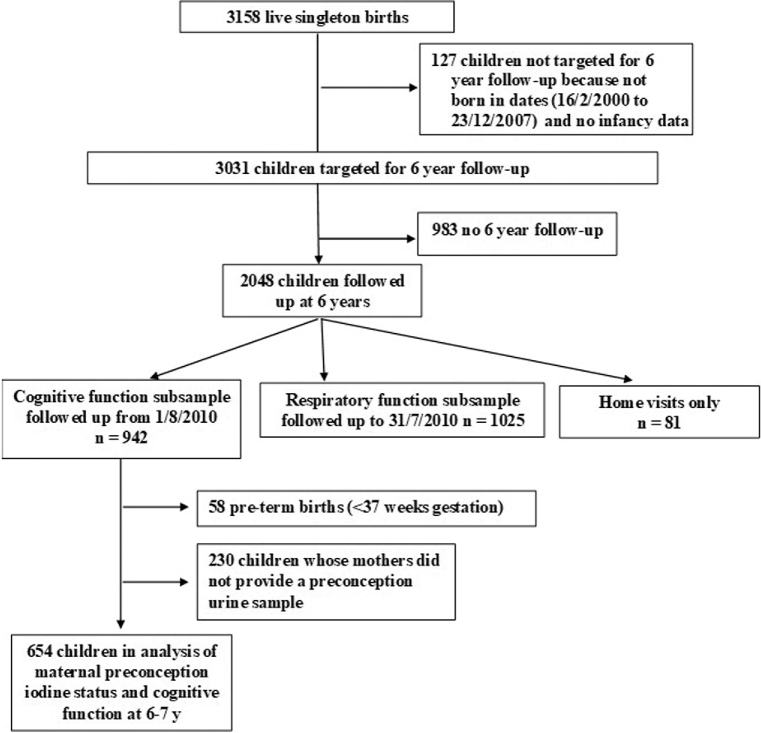
Flow chart showing the 654 children assessed at 6–7 y in the Southampton Women's Survey.

From August 2010, trained research nurses administered a variety of tests of cognitive function at the 6–7 y home visit, supervised by an educational psychologist; there were 942 children in this subgroup (**Figure 1**). Full-scale IQ—an estimate of general cognitive ability—was assessed using the 2-subtest form of the Wechsler Abbreviated Scale of Intelligence (WASI) (22). This scale has been shown to have excellent reliability, with an average reliability coefficient (internal consistency) for full-scale IQ of 0.93 and average interrater agreement >0.90 for verbal and nonverbal subtests. In the present study, interrater reliability statistics, determined from comparison of nurse-assessed video interviews, were very similar (>0.9). Specific components of executive function were assessed using tests from the Cambridge Neuropsychological Test Automated Battery (CANTAB, Cambridge Cognition, Cambridge, United Kingdom) (23). Delayed Matching to Subject (DMS) and Spatial Span (SSP) are both tests of memory. DMS assesses forced choice recognition memory for novel nonverbalizable patterns, and tests both simultaneous and short-term visual memory. SSP assesses working memory capacity. Intra-Extra Dimensional Set Shift (IED) is a test of rule acquisition and reversal that assesses the ability to engage in deliberate, goal-directed action. Several outcome measures are available for each CANTAB test. In the current analysis, to reduce the likelihood of chance findings, and consistent with earlier analyses (23), we used the following outcomes: DMS, total correct (12-s delay); IED, total errors (adjusted for each stage not attempted due to failure); and SSP, span length (longest sequence successfully recalled). Maternal intelligence (full-scale IQ) was also assessed, using the WASI (22).

#### Statistical analysis

Of the children who had an assessment of cognitive function when they were aged 6–7 y, 58 were excluded as they were born preterm (<37 wk of gestation). Of the remaining 884 children, 654 (74%) were born to mothers who had provided preconception urine samples (Figure 1). Iodine status (I/Cr) was used as a continuous variable throughout the analyses, but categorized for presentation. I/Cr values and CANTAB IED total errors (adjusted) values were positively skewed and therefore transformed using Fisher-Yates normal scores (24). Full-scale IQ (WASI), CANTAB DMS total correct (12-s delay), and CANTAB SSP span length were standardized for analyses so the results could be interpreted in terms of SD changes; Pearson correlations were used to compare these measures. Linear regression models were used to assess the association between I/Cr and cognitive function as measured by full-scale IQ (WASI) and executive function (CANTAB); the analyses are presented unadjusted and adjusted for confounders. The choice of confounders was informed by a Directed Acyclic Graph (**Supplemental Figure 1**); this suggested adjusting for maternal IQ, maternal education, and maternal prepregnancy BMI. In addition, breastfeeding duration, smoking in pregnancy (yes or no), sex, age, and maternal iodine intakes in pregnancy were included to improve the accuracy of the model. Only the CANTAB outcomes were adjusted for age in the analysis, as the derivation of full-scale IQ includes adjustment for age. A sample size of 422 participants (the smallest number included in the fully adjusted models) would have 80% power to detect a regression coefficient of 0.135 on the measured cognitive outcome for a 1 SD increase in the iodine:creatinine ratio, at a 5% significance level. The data were analyzed using Stata version 14.1 (StataCorp LP).

## Results

### 

#### Participant characteristics

The characteristics of 654 mothers and children in the subsample of SWS for whom preconception maternal iodine status and cognitive function data were available are shown in **Table 1**, together with data for the remaining SWS mothers and children (born at term) who were not included in the analyses. In comparison with the rest of the cohort, the mothers in the subsample were of similar age and BMI. The majority of women in both groups did not take iodine-containing supplements in the preconception period. However, there were some differences when women in the subsample were compared with the rest of the cohort: they had slightly lower iodine intakes, were less likely to smoke in pregnancy, and more likely to have breastfed for longer (all *P *< 0.05). There was also a difference in average maternal IQ between the 2 groups; in the subsample, maternal IQ was higher and a greater proportion of mothers had educational qualifications at least to 18 y (both *P *< 0.05). Children in the subsample did not differ in weight at birth or duration of gestation when compared with the rest of the SWS children and the proportion of boys was similar.

**TABLE 1:** Characteristics of the 654 mother-child pairs studied and the rest of the SWS cohort^1^

	Analysis sample (*n = *654)	Rest of SWS cohort (*n = *2297)	*P* value for difference
Maternal characteristics			
Age at preconception assessment, y	27.5 ± 3.8^2^	27.8 ± 3.9	0.11
Preconception BMI, kg/m^2^	24.3 (21.9–26.9)^3^	24.1 (21.9–27.5)	0.82
Total iodine intake, µg/d	147 (116–193)	155 (116–210)	0.01
Women taking iodine supplements in preconception period, %	12	10	0.12
Women who smoked:			
In preconception period, %	24	29	0.007
In pregnancy, %	10	18	<0.001
Women with qualifications to at least A-level,^4^ %	66	57	<0.001
Maternal IQ^5,6^	108.4 ± 12.6	105.0 ± 13.3	0.02
Child characteristics			
Boys, %	51	52	0.85
Gestational age at birth, wk	40.2 ± 1.2	40.1 ± 1.2	0.05
Birthweight, kg	3.5 ± 0.5	3.5 ± 0.5	0.84
Duration of breastfeeding, wk	13.0 (2.0–30.4)	8.7 (0.4–26.1)	<0.001
Age at assessment of cognitive function, y	6.9 ± 0.2	—	
Full-scale IQ	103.7 ± 15.3	—	
DMS, total correct (12-s delay)	2.6 ± 1.2	—	
IED, total errors (adjusted)	58 (37–63)	—	
SSP, span length	3.9 ± 0.9	—	

^1^Analysis sample comprised children who had measured cognitive function at 6–7 y and whose mother's iodine status was assessed. CANTAB outcomes: DMS, IED, SSP. CANTAB, Cambridge Neuropsychological Test Automated Battery; DMS, Delayed Matching to Sample; IED, Intra-Extra Dimensional Set Shift; SSP, Spatial Span; SWS, Southampton Women's Survey.

^2^Mean ± SD (all such values).

^3^Median; IQR in parentheses (all such values).

^4^School examinations taken at age 18 y.

^5^Maternal intelligence assessed when children were aged 6–7 y using the Wechsler Abbreviated Scale of Intelligence (22).

^6^Data available for 243 women in the “rest of SWS cohort” group.

#### Maternal iodine status

**FIGURE 2:**
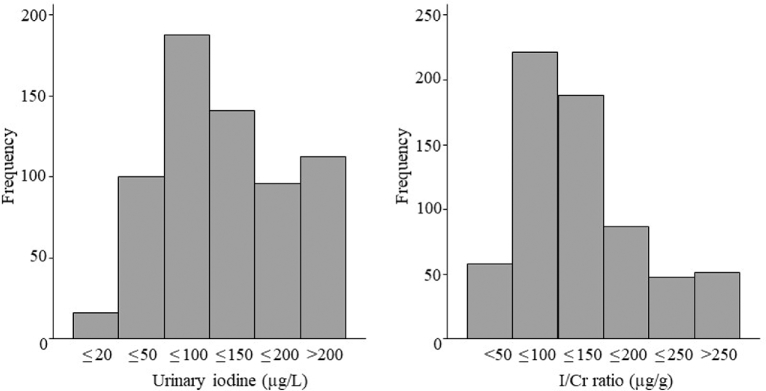
Frequency distributions of preconception urinary iodine concentration and I/Cr ratio, determined in spot samples from 654 women in the Southampton Women's Survey. I/Cr, iodine:creatinine.

**Figure 2** shows the distributions of maternal UIC and the I/Cr. Median I/Cr was 114 µg/g (IQR: 76–164 µg/g); median UIC was 108.4 µg/L (IQR: 62.2–167.8 µg/L). 17.8% of women had a preconception UIC <50 µg/L. The timing of urine collections ranged between 0900 and 2000, although the majority (85%) of samples were collected after 1200. UIC was inversely related to time of sample collection (*P = *0.018), but there was no association between time of sample collection and I/Cr (*P = *0.87). We therefore did not consider time of collection of sample further in the analyses of I/Cr data.

The characteristics of mothers and children studied are shown in **Supplemental Table 1**, according to maternal preconception iodine status (I/Cr). Maternal iodine status was positively related to age and inversely related to preconception BMI, although the differences were small. As expected, iodine status was positively related to iodine intake and to use of iodine-containing supplements; almost a fifth of women who had an I/Cr ≥150 µg/g were taking iodine-containing supplements. There were no differences in maternal educational attainment or IQ in relation to iodine status in the preconception period. No differences were found in the weight of the infant or gestational age at birth in relation to maternal iodine status; higher iodine status tended to be associated with longer duration of breastfeeding but this did not achieve statistical significance.

#### Preconception iodine status and cognitive function in childhood

**FIGURE 3:**
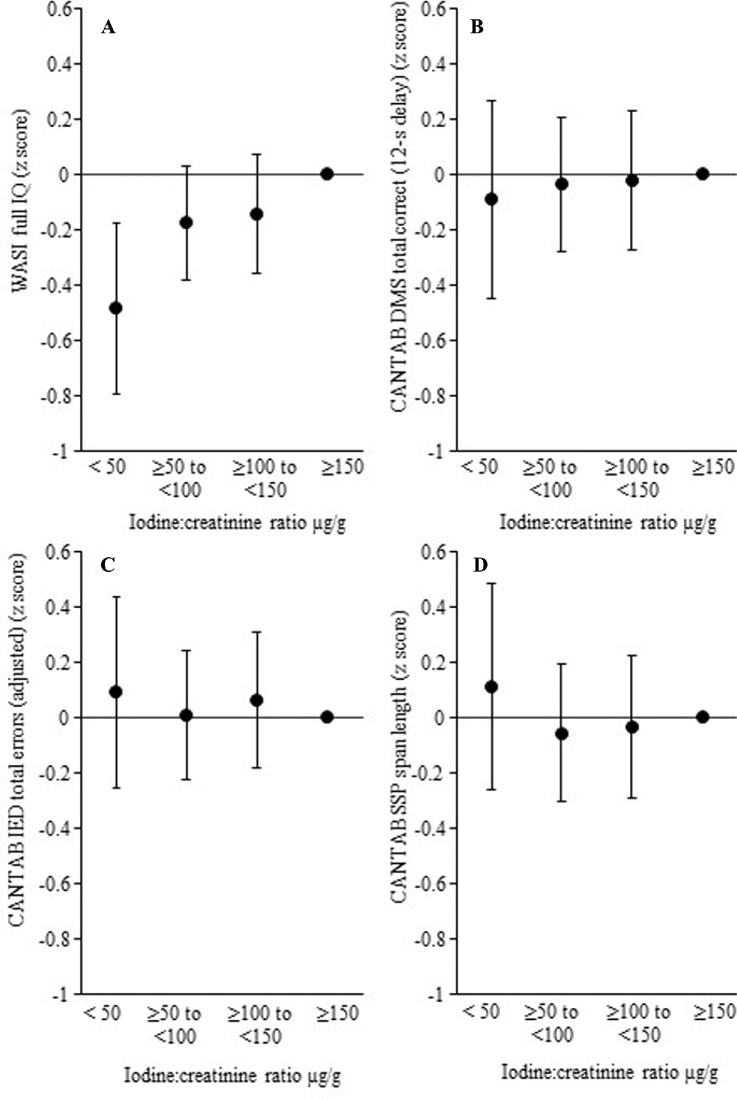
Adjusted differences in cognitive function at 6–7 y in 654 chidren in the Southampton Women's Survey, according to maternal preconception iodine status (iodine:creatinine ratio). Numbers of mother-child pairs—iodine:creatinine ratio: <50, *n* = 58; ≥50 to <100, *n* = 222; ≥100 to <150, *n* = 188; ≥150, *n* = 186. Cognitive function assessed using the WASI (22) (A) and Cambridge Neuropsychological Test Automated Battery (CANTAB, Cambridge Cognition, Cambridge, United Kingdom) (23) (B–D); data adjusted for maternal IQ, maternal education, prepregnancy BMI, duration of breastfeeding, smoking in pregnancy, sex, and age (for CANTAB outcomes); reference group iodine:creatinine ratio: ≥ 150µg/g; values are means and 95% CIs. IED adjusted by adding 25 for each stage not attempted due to failure. CANTAB, Cambridge Neuropsychological Test Automated Battery; DMS, Delayed Matching to Sample; IED, Intra-Extra Dimensional Set Shift; SSP, Spatial Span; WASI, Wechsler Abbreviated Scale of Intelligence.

**Table 2** shows the associations between maternal preconception iodine status (I/Cr) and children's cognitive function outcomes at the age of 6–7 y, before and after adjustment for potential confounders. Preconception iodine status was positively associated with the child's full-scale IQ; this association was robust to adjustment for the potential confounding influences including adjustment for the effects of maternal IQ. When we included quadratic terms into the adjusted model, we found no evidence of nonlinearity in this association (data not shown). Full-scale IQ was not strongly correlated with DMS, total correct (12-s delay) (*r* = 0.10, *P* = 0.03); IED, total errors (adjusted) (*r* = −0.16, *P* < 0.001); or SSP, span length (*r* = 0.27, *P* < 0.001). In contrast to the association seen with full-scale IQ, maternal iodine status was not related to any of the executive function outcomes assessed using CANTAB—either before or after adjustment for confounders. The contrasting associations between maternal preconception iodine status and child IQ and assessed executive function are illustrated in **Figure 3**; the nonstandardized IQ data are shown in **Supplemental Figure 2**. Preconception urinary I/Cr was <50 µg/g in 8.9% of women; compared with those with an I/Cr ≥150 µg/g, their offspring IQ was 0.49 (95% CI: 0.79, 0.18) SD lower, equivalent to a difference of 7.5 points (Supplemental Figure 2).

**TABLE 2:** Maternal preconception iodine status (iodine:creatinine ratio) as a predictor of cognitive function at age 6–7 y in the 654 mother-child pairs studied^1^

	Unadjusted			Adjusted^2^		
	β (95% CI)	*P* value	*n*	β (95% CI)	*P* value	*n*
Full scale IQ (*z* score)	0.09 (0.02, 0.17)	0.02	647	0.13 (0.04, 0.21)	0.003	507
Executive function						
CANTAB DMS total correct (12-s delay) (*z* score)	0.03 (−0.06, 0.11)	0.52	540	0.01 (−0.09, 0.10)	0.90	453
CANTAB IED total errors (adjusted^3^) (*z* score)	−0.07 (−0.15, 0.02)	0.12	538	−0.04 (−0.14, 0.05)	0.36	451
CANTAB SSP span length (*z* score)	0.00 (−0.08, 0.09)	0.95	505	0.00 (−0.10, 0.09)	0.93	422

^1^Cognitive function assessed using the Wechsler Abbreviated Scale of Intelligence (full-scale IQ) (22) and Cambridge Neuropsychological Test Automated Battery (CANTAB, Cambridge Cognition, Cambridge, United Kingdom) (23). Iodine:creatinine ratio (*z* score). β Values represent the slope of association between maternal iodine status and children's cognitive outcomes. CANTAB, Cambridge Neuropsychological Test Automated Battery; DMS, Delayed Matching to Sample; IED, Intra-Extra Dimensional Set Shift; SSP, Spatial Span.

^2^Adjusted for maternal IQ, maternal education, prepregnancy BMI, duration of breastfeeding, smoking in pregnancy, sex, and age (for CANTAB outcomes).

^3^Adjusted by adding 25 for each stage not attempted due to failure.

Because iodine status was assessed before conception, we also considered changes in iodine intakes in pregnancy from the preconception period (**Supplemental Table 2**). There was a small increase in the use of iodine-containing supplements in early pregnancy. However, the more notable change was an overall increase in dietary iodine intake in early pregnancy (*P < *0.001) followed by a further increase in late pregnancy (*P < *0.001). These changes were partly explained by an increase in milk intake in pregnancy, as we have previously reported (19). The proportion of women who had iodine intakes below the UK Reference Nutrient Intake (RNI) (140 µg/d) fell from almost half the group before conception to less than a third by late pregnancy. In the next analyses we therefore further adjusted the models for differences in maternal iodine intake in pregnancy. The findings were largely unchanged: maternal preconception iodine status was still positively associated with child IQ, but was not related to any of the executive function outcomes examined (**Supplemental Table 3**).

Our final analyses considered the timing of assessment of preconception iodine status. As it is possible that more distant assessments may be a weaker marker of iodine status at conception, we tested for interactions between preconception iodine status and time to conception; we found no evidence of differential effects (**Supplemental Table 4**).

## Discussion

In a general population sample of UK women, lower iodine status before conception was related to lower IQ in their child at the age of 6–7 y. The association provides some support for links between maternal iodine status in pregnancy and child IQ that have been previously reported (14), and importantly, we found the association to be robust to adjustment for a range of potential confounding factors, including maternal IQ. To our knowledge, associations between iodine status in the preconception period and children's cognitive function have not been reported before. However, in light of the reported links between maternal iodine insufficiency in pregnancy and children's school performance (14, 15), an unexpected finding of our study was that there were no associations between maternal iodine status in the preconception period and measures of executive function.

Although mild to moderate iodine deficiency is common in many parts of the world, clear evidence of the effects of maternal insufficiency on child neurodevelopment is lacking (25). Recent observational studies have started to address this gap in knowledge. They show deficits in child IQ and school performance (14, 15) in relation to lower maternal iodine status in pregnancy, and, most recently, child language delay and behavior problems at the age of 3 y among children born to women who had low iodine intakes in pregnancy (16). Although we cannot compare our preconception data directly with the findings from studies of pregnant women, the variations in iodine status we describe across our study population are very comparable to other UK studies. For example, the median UIC in our study was 108 µg/L, which compares with values of 91 µg/L in the Avon Longitudinal Study of Parents and Children (ALSPAC) cohort in pregnancy (14) and 117 µg/L among a national sample of nonpregnant women of childbearing age in the recent data collection in the NDNS (7). A median UIC value >100 µg/L in our study, together with the findings that 18% of SWS women had UIC values <50 µg/L, would categorize this group as having adequate iodine intake, using WHO criteria (6). Consistent with these indications of iodine sufficiency, median iodine intakes were close to the RNI in the preconception period. Furthermore, intakes increased in line with greater milk consumption in pregnancy (Supplemental Table 2), such that less than a third of women had intakes below the RNI by late pregnancy. However, despite these positive indications of iodine sufficiency, some of the women studied had low iodine status, and lower status was related to sizeable deficits in child IQ when assessed at 6–7 y. In terms of effect size, the differences in child IQ (Supplemental Figure 2) appear greater than the differences observed in the ALSPAC cohort, which did not take account of differences in maternal IQ (14). But it is important to note that the group of SWS women who had I/Cr <50 µg/g was relatively small in our study, and the CIs were wide.

One interpretation of our findings is that maternal iodine status was insufficient to support optimal fetal neurodevelopment for some women in the study population. Iodine is required for the synthesis of thyroid hormones and adverse effects of maternal hypothryoxinemia on fetal brain development are known (13), although this relation may be complex as both low and high maternal free thyroxine concentrations have been linked to deficits in child IQ (26). However, it is also important to highlight the negative findings from our study; contrary to expectation, we found no differences in other measures of cognitive ability, namely scores on 3 tests of executive function. Executive function describes various cognitive processes that coordinate, monitor, and maintain other more basic cognitive processes involved in learning, reasoning, and goal-directed behavior. Measured executive function has been shown to be associated with better performance in tests of reading and math in childhood (27). There is evidence that measured executive function is associated with intelligence although, as in the present study, correlations between scores are modest (28). Given the demonstrated links between low maternal iodine status and poorer school performance in other studies that point to negative effects on working memory capacity and visual processing skills (15, 29), we would have expected to see patterns of associations comparable with those we observed for child IQ. It is not clear why the associations with different measures of cognitive function were not consistent, and further data are needed. One possibility is that the variability in the CANTAB measures selected for analysis was modest. Although we had an a priori analysis plan, that included a specified set of outcomes, and we used a directed acyclic graph approach to identify confounding factors to consider, these are observational data and we cannot exclude the possibility of residual confounding, or that the single association we found between iodine status and child IQ was a chance finding. However, our data add to earlier evidence (14) that also showed links between lower maternal iodine status and lower offspring IQ; the consistency of findings raises concerns.

A strength of this study is that we studied a large general population sample of women and children; the women had been characterized in detail in the period before conception, and we collected data on a range of measures of their child's cognitive function at age 6–7 y. But the study also has some limitations. First, we defined iodine status using single spot urine samples. Although spot samples are commonly used to describe iodine status in population studies, and are considered to provide a good index of iodine intake over the past day, 24-h samples and/or repeat spot samples are recommended for individual studies to take account of variations in iodine excretion (30, 31). Our analyses therefore used the I/Cr in order to correct for differences in urine volume; I/Cr has been demonstrated to have better agreement with 24-h iodine excretion than UIC (32, 33). A second limitation is that for some women in the group, significant time had elapsed between the date of the urine sample and the date of conception. We have previously described stability in dietary habits in the preconception period in this population, such that the women's dietary patterns when reassessed after 2 y were very similar (correlation between “prudent” dietary pattern scores = 0.81; average change in score = 0.13 SD) (34). As these data suggest that iodine status would not have changed markedly by the time women conceived, the analyzed samples should provide a reasonable indication of iodine status at conception. However, although we found no evidence of differential effects according to length of time between assessment of iodine status and conception, it is possible that changes had occurred. A third limitation is that we studied a subgroup of women who were taking part in the SWS, and some characteristics, such as maternal IQ and educational attainment, differed from those women who were not included in the analyses. This could affect the generalizability of our findings, although the variations in iodine status of the SWS women that we describe are comparable to recent status data from a nationally representative sample of women of childbearing age (7). A final limitation is that we did not collect urine samples from the children. As we are unable to take account of differences in iodine status in childhood, we cannot exclude the possibility of postnatal effects of differences in status on cognitive function.

The benefits of iodine supplementation for mildly deficient women are uncertain (2), and further data are needed (25, 35). The high rates of unplanned pregnancy in the United Kingdom (36), together with the known vulnerability of the fetus to the effects of iodine deficiency in early gestation, may limit the effectiveness of iodine supplementation starting in pregnancy, suggesting that the time to optimize iodine status is before conception. In addition, a recent analysis from the Norwegian Mother and Child Cohort Study (16) showed no protective effects of iodine supplementation during pregnancy on offspring neurodevelopment; there was also some suggestion of harmful effects of supplementation among women who had low dietary iodine intakes (16). Although ongoing research will be key to defining the prevalence and public health importance of low iodine status, our study adds further data to a body of evidence that focuses attention on the importance of nutrition in the preconception period.

## Supplementary Material

Supplemental data

## Notes

Supported by the Medical Research Council, British Heart Foundation, Food Standards Agency, Arthritis Research UK, and NIHR Southampton Biomedical Research Centre. The research leading to these results has also received funding from the European Union's Seventh Framework Programme (FP7/2007-2013), project EarlyNutrition under grant agreement n°289346. KMG, SMR, PCC, and CC are supported by the National Institute for Health Research through the NIHR Southampton Biomedical Research Centre.

Author disclosures: KMG has received reimbursement for speaking at conferences sponsored by companies selling nutritional products and is part of an academic consortium that has received research funding from Abbot Nutrition, Nestec, and Danone. SMR, SRC, EAM, CRG, PCC, CC, and HMI, no conflicts of interest.

Supplemental Tables 1–4 and Supplemental Figures 1 and 2 are available from the “Supplementary data” link in the online posting of the article and from the same link in the online table of contents at https://academic.oup.com/jn/.

Abbreviations used: CANTAB, Cambridge Neuropsychological Test Automated Battery; DMS, Delayed Matching to Sample; I/Cr, iodine:creatinine ratio; IED, Intra-Extra Dimensional Set Shift; NDNS, National Diet and Nutrition Survey; RNI, Reference Nutrient Intake; SSP, Spatial Span; SWS, Southampton Women's Survey; UIC, urinary iodine concentration; WASI, Wechsler Abbreviated Scale of Intelligence.
