# Supplementary material for: Preconception Maternal Iodine Status Is Positively Associated with IQ but Not with Measures of Executive Function in Childhood
Source: J Nutr. 2018 May 15;148(6):959–66. doi: 10.1093/jn/nxy054 (PMC5991217; doi:10.1093/jn/nxy054)
Supplement: Supplemental data [file nxy054_supplemental_files.docx]

**Supplemental Figure 1. Directed acyclic graph**

**
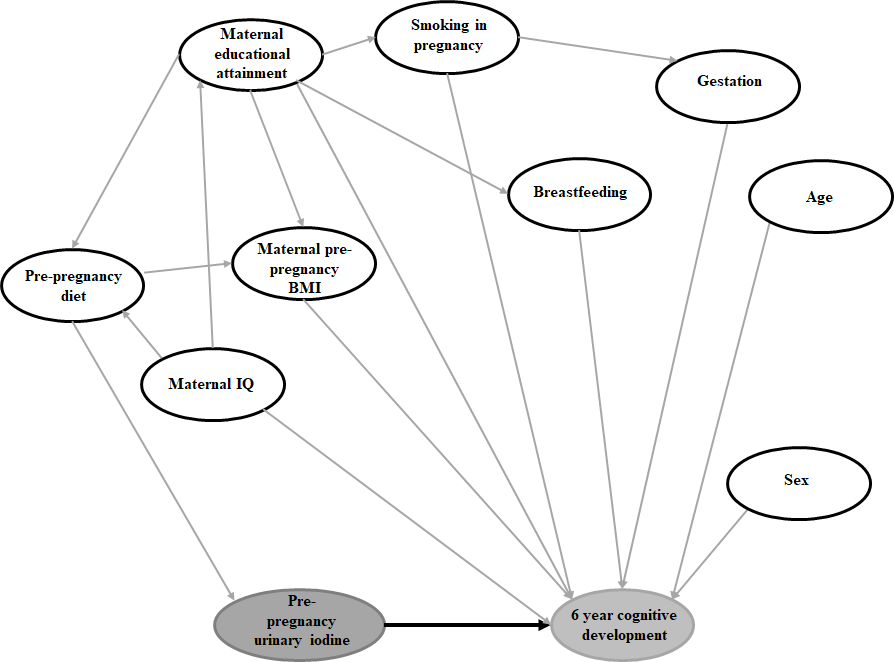
**

**Supplemental Figure 2. Adjusted differences in IQ at 6-7 years according to maternal preconception iodine status (iodine:creatinine ratio)^1,2,^**

-15

-10

-5

0

5

WASI full IQ

< 50

≥50 to

< 100

≥ 150

Iodine:creatinine ratio µg/g

≥100 to

< 150

^1^IQ assessed using the Wechsler Abbreviated Scale of Intelligence (full-scale IQ) [22]; ^2^adjusted for maternal IQ, maternal education, pre-pregnancy BMI, duration of breastfeeding, smoking in pregnancy and sex; numbers of mother-child pairs: I/Cr <50, n=58; ≥ 50 to < 100, n=222; ≥ 100 to < 150, n=188; ≥ 150, n=186; reference group I/Cr ≥150µg/g; Values are means and 95% confidence intervals.

**Supplemental Table 1. Maternal and child characteristics according to maternal preconception iodine status (iodine:creatinine ratio)**

|  | **Preconception urinary iodine to creatinine ratio (μg/g)^1^** | | | |  |
| --- | --- | --- | --- | --- | --- |
|  | **< 50** | **≥ 50 to < 100** | **≥ 100 to < 150** | **≥ 150** |  |
| **Maternal characteristics** | ***(n = 58)*** | ***(n = 222)*** | ***(n = 188)*** | ***(n = 186)*** | ***P*-value^2^** |
|  |  |  |  |  |  |
| Age at preconception assessment (years)^3^ | 26.1 ± 3.9 | 27.0 ± 3.9 | 28.0 ± 3.5 | 27.9 ± 3.8 | < 0.001 |
|  |  |  |  |  |  |
| Preconception BMI (kg/m^2^)^4^ | 24.8 (21.6, 27.7) | 24.5 (22.6, 27.2) | 24.8 (22.1, 27.0) | 23.6 (21.5, 26.0) | 0.001 |
|  |  |  |  |  |  |
| % women taking iodine- containing supplements in preconception period (%) | 5 | 8 | 12 | 18 | < 0.001 |
| Preconception total iodine intake (μg/day)^4^ | 144 (110, 183) | 137 (109, 187) | 155 (117, 193) | 157 (122, 213) | 0.002 |
|  |  |  |  |  |  |
| % women who smoked: |  |  |  |  |  |
| - preconception (%) | 24 | 23 | 22 | 26 | 0.55 |
| - in pregnancy (%) | 11 | 7 | 9 | 14 | 0.02 |
|  |  |  |  |  |  |
| % women with qualifications to at leastA-level (%)^5^ | 83 | 62 | 70 | 62 | 0.24 |
|  |  |  |  |  |  |
| Maternal IQ^3,6^ | 108.8 ± 11.6 | 108.4 ± 12.8 | 107.4 ± 13.0 | 109.4 ±12.0 | 0.91 |
| **Child characteristics** |  |  |  |  |  |
| Gestational age at birth (weeks)^3^ | 40.1 ± 1.2 | 40.2 ± 1.2 | 40.0 ± 1.2 | 40.3 ± 1.3 | 0.98 |
|  |  |  |  |  |  |
| Birthweight (kg)^3^ | 3.4 ± 0.5 | 3.5 ± 0.5 | 3.5 ± 0.5 | 3.5 ± 0.5 | 0.56 |
|  |  |  |  |  |  |
| Duration of breastfeeding (weeks)^4^ | 8.7 (3.0, 21.7) | 15.0 (2.0, 32.3) | 10.7 (1.0, 29.0) | 15.4 (3.0, 34.8) | 0.15 |

^1^determined on spot samples; ^2^test for continuous association from regression model (not mutually adjusted); ^3^mean ± SD and all such values; ^4^median (interquartile range) and all such values; ^5^school examinations taken at 18 years; ^6^maternal intelligence assessed when children were aged 6-7 years using the Wechsler Abbreviated Scale of Intelligence [22].

**Supplemental Table 2. Iodine intakes in preconception period and during pregnancy of 654 women in the Southampton Women’s Survey, whose children had cognitive assessments at 6-7 years of age**

|  | **Preconception** | **Early pregnancy^1^** | **Late pregnancy^2^** |
| --- | --- | --- | --- |
|  |  |  |  |
|  |  |  |  |
| Dietary iodine intake (µg/day)^3^ | 139 (111, 183) | 152 (119, 191) | 171 (136, 217) |
|  |  |  |  |
| % women taking iodine-containing supplements (%) | 12 | 16 | 13 |
|  |  |  |  |
| - users’ supplementary  iodine intake (µg/day)^3^ | 83 (33, 135) | 85 (47, 138) | 124 (65, 140) |
|  |  |  |  |
| Total iodine intake (µg/day)^3^ | 147 (116, 193) | 161 (125, 209) | 179 (138, 235) |
|  |  |  |  |
| % women with total iodine intake < 140 µg/d^4^ | 45 | 34 | 27 |
|  |  |  |  |
| % women with total iodine intake < 70 µg/d^5^ | 3 | 2 | 2 |

^1^assessed at 11 weeks’ gestation; ^2^assessed at 34 weeks’ gestation; ^3^median (interquartile range) and all such values; ^4^UK reference nutrient intake; ^5^UK lower reference nutrient intake.

**Supplemental Table 3. Maternal preconception iodine status (iodine:creatinine ratio) as a predictor of cognitive function at age 6-7 years^1,2^ after adjustment for confounding factors and maternal iodine intake in pregnancy**

|  |  | | |
| --- | --- | --- | --- |
|  | **Beta (95% CI)^3^** | ***P*-value** | ***n*** |
|  |  |  |  |
| Full-scale IQ (z-score) | 0.12 (0.02, 0.22) | 0.02 | *348* |
| *Executive function* |  |  |  |
| CANTAB DMS total correct (12 sec delay) (z-score) | -0.05 (-0.17, 0.06) | 0.36 | *314* |
|  |  |  |  |
| CANTAB IED total errors (adjusted^4^)  (z-score) | -0.05 (-0.16, 0.06) | 0.37 | *314* |
|  |  |  |  |
| CANTAB SSP span length (z-score) | -0.05 (-0.17, 0.07) | 0.43 | *295* |
|  |  |  |  |

^1^Cognitive function assessed using the Wechsler Abbreviated Scale of Intelligence (full-scale IQ) [22] and Cambridge Neuropsychological Test Automated Battery (CANTAB®, Cambridge Cognition, Cambridge, UK) [23]; ^2^Iodine:creatinine ratio (z-score); ^3^adjusted for maternal IQ, maternal education, pre-pregnancy BMI, duration of breastfeeding, smoking in pregnancy, maternal iodine intake in early pregnancy, sex and age (for CANTAB outcome); ^4^adjusted by adding 25 for each stage not attempted due to failure.

**Supplemental Table 4. P-value for interaction between maternal preconception iodine status (iodine:creatinine ratio) and time to conception on cognitive function at age 6-7 years**

|  | **Unadjusted** | **Adjusted^1^** |
| --- | --- | --- |
|  |  |  |
| Full scale IQ (z-score) | *P* = 0.91 | *P* = 0.23 |
| CANTAB DMS total correct (12 sec delay) (z-score) | *P* = 0.88 | *P* = 0.66 |
| CANTAB IED total errors (adjusted) (z-score) | *P* = 0.68 | *P* = 0.51 |
| CANTAB SSP span length (z-score) | *P* = 0.54 | *P* = 0.28 |

^1^Adjusted for maternal IQ, maternal education, pre-pregnancy BMI, duration of breastfeeding, smoking in pregnancy, sex and age (for CANTAB outcome);
